# In Vitro Analyses of the Multifocal Effects of Natural Alkaloids Berberine, Matrine, and Tabersonine against the O’nyong-nyong Arthritogenic Alphavirus Infection and Inflammation

**DOI:** 10.3390/ph16081125

**Published:** 2023-08-09

**Authors:** Anne-Laure Sandenon Seteyen, Pascale Guiraud, Philippe Gasque, Emmanuelle Girard-Valenciennes, Jimmy Sélambarom

**Affiliations:** 1 Unité de Recherche Etudes Pharmaco-Immunologiques (UR-EPI), Université de La Réunion, 97400 Saint-Denis, France; anne-laure.sandenon-seteyen@univ-reunion.fr (A.-L.S.S.); pascale.guiraud@univ-reunion.fr (P.G.);; 2 Centre Hospitalier Universitaire de La Réunion, Laboratoire d’Immunologie Clinique et Expérimentale de la Zone Océan Indien (LICE-OI), Pôle de Biologie, 97400 Saint-Denis, France; 3 Laboratoire de Chimie et de Biotechnologie des Produits Naturels (CHEMBIOPRO), Université de La Réunion, 97400 Saint-Denis, France

**Keywords:** O’nyong-nyong virus, natural products, alkaloids, antiviral, anti-inflammatory

## Abstract

O’nyong-nyong virus (ONNV) is a member of the reemerging arthritogenic alphaviruses that cause chronic debilitating polyarthralgia and/or polyarthritis via their tropism for the musculoskeletal system. Thus, the discovery of dual antiviral and anti-inflammatory drugs is a great challenge in this field. We investigated the effects of the common plant-derived alkaloids berberine (isoquinoline), matrine (quinolizidine), and tabersonine (indole) at a non-toxic concentration (10 μM) on a human fibroblast cell line (HS633T) infected by ONNV (MOI 1). Using qRT-PCR analyses, we measured the RNA levels of the gene coding for the viral proteins and for the host cell immune factors. These alkaloids demonstrated multifocal effects by the inhibition of viral replication, as well as the regulation of the type-I interferon antiviral signaling pathway and the inflammatory mediators and pathways. Berberine and tabersonine proved to be the more valuable compounds. The results supported the proposal that these common alkaloids may be useful scaffolds for drug discovery against arthritogenic alphavirus infection.

## 1. Introduction

Alphaviruses belong to the enveloped single-stranded positive RNA viruses of the *Togaviridae* family; they are globally reemerging arthropod-borne viruses (arboviruses) transmitted to mammalians by mosquitoes. They typically cause fever, rash, and/or myalgia [1,2,3]. Alphavirus genomes mainly consist of genes that code for a capsid protein (C), two envelope glycoproteins (E1/E2), and four non-structural proteins (nsP1-nsp4) [4]. O’nyong-nyong virus (ONNV) was first isolated during a major epidemic in East Africa in 1959–1962, and it spread in West Africa with two other outbreaks in 1996 and 2002 [5]. ONNV is a member of the Old World alphaviruses (vs. the New World alphaviruses), with Barmah Forest virus (BFV), Chikungunya virus (CHIKV), Ross River virus (RRV), Mayaro virus (MAYV), Sindbis virus (SINV), and Semliki Forest virus (SFV) [6,7]. The latter are referred to as arthritogenic alphaviruses since they are characterized by the debilitating polyarthralgia and/or polyarthritis resulting from their tropism for muscle, bone, and joint tissue and the resulting cell inflammatory infiltration [8,9,10,11]. Fibroblasts are the main target cells during alphavirus infection [12,13,14]. Formerly described as structural cells, fibroblasts are now well established as immunocompetent cells that detect pathogens and activate defensive pathways [15].

During infection, the host immune system operates via a complex array of mechanisms for viral detection and clearance, as well as for the protective inflammatory response that is normally regulated to prevent the aforementioned disorders [16]. After the granting of viral entry by the receptors, especially matrix remodeling-associated 8 (MxRa8) [17], the innate immune antiviral response and pattern recognition receptors (PRRs) are engaged. Viral RNA (single-stranded) can be detected by the cytosolic retinoic acid-inducible gene I (RIG-I)-like receptors (RLRs) to promote the expression of various host anti-alphaviral defenses, including the essential type-I interferons (IFNs). Hence, the downstream signals from the PRRs involve the activation of interferon regulatory factors (IRFs), leading to the production of IFNs and, subsequently, the signal transducer and activator of transcription (STAT)-dependent production of interferon-stimulated genes (ISGs) [18]. The immune response is achieved by a range of inflammatory mediators for the recruitment of other defensive cells and/or the infiltration of immune cells that may cause severe inflammation. The critical pro-inflammatory mediators include the monocyte chemoattractant protein-1 (MCP-1/CCL2) [19]; the T helper type 1 (Th1)-cytokines tumor necrosis factor alpha (TNF-α) and interleukin-8 (IL8/CXCL8) [20]; the CCL5 chemokine, also known as RANTES (Regulated upon Activation, Normal T cell Expressed, and Secreted) [21]; and prostaglandin PGE2 [22]. The overall immune response against alphaviruses is consistent with the activation of several canonical inflammatory pathways, including those related to mitogen-activated protein kinase (MAPK); the nuclear factor kappa-B (NF-κB); the Janus kinase/signal transducer and activator of transcription (JAK-STAT) [23]; the phosphatidylinositol 3-kinase/protein kinase B (PI3K/AKT) [24]; or cyclooxygenase-2 (COX-2) [22]. Additionally, the NOD-like receptor family, pyrin domain-containing 3 (NLRP3) inflammasome may trigger caspase-1 activation and, consequently, the production of the highly inflammatory IL-1β [25,26].

The management of arthritogenic alphavirus infection currently consists of symptom relief by conventional analgesics (e.g., paracetamol) and non-steroidal anti-inflammatory drugs (NSAIDs), such as aspirin, during the acute phase or alleviation of arthritic disorders during the chronic stage using disease-modifying antirheumatic drugs (DMARDs) such as methotrexate [27,28]. Despite continuous research efforts, there are currently no specific treatment or vaccines against alphavirus infection [29,30].

Alkaloids are nitrogen-containing secondary metabolites, mainly found in plants. They display a wide range of biological activities, including, notably, those against the most prominent human health disorders (e.g., cancer, infection, or inflammation) [31]. Through computational studies, plant-derived alkaloids have been identified as potential nsP2 protease inhibitors of various alphaviruses [32]. With regard to the most frequently studied CHIKV infection, in vitro studies established the inhibitory effects of the cephalotaxine alkaloid harringtonine on viral replication [33], the isoquinoline berberine on the nucleocapsid assembly [34], the phenanthridine-derived lycorine on the viral translation [35], and the steroidal alkaloid tomatidine on both the viral replication and viral protein translation [36].

**Figure 1:**
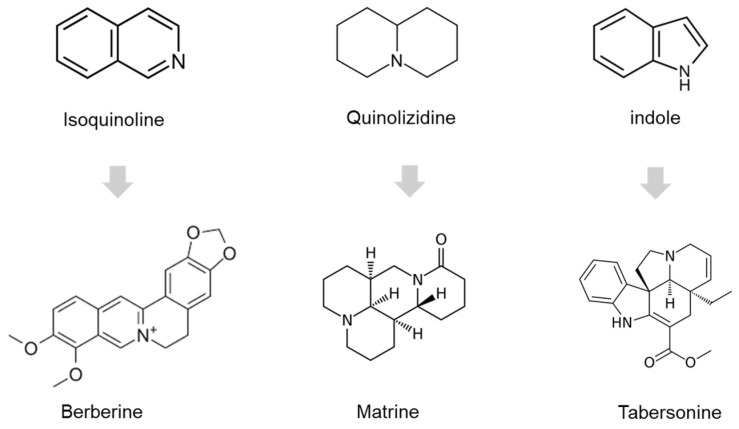
Chemical structures.

In addition to this common antiviral strategy based on virus-targeting inhibitors (‘direct-acting antivirals’) or host-targeting inhibitors to abrogate the essential host cell functions for the virus life cycle (‘host-targeting antivirals’), immunomodulatory drugs have gained growing interest as regulators of the immune response [37,38]. In primary human synovial fibroblast cells (HSF) infected by ONNV, we demonstrated the antiviral and immunomodulatory activity of the anticancer drug Irinotecan (CPT-11), derived from the natural quinoline alkaloid camptothecin [39]. As we recently discussed in a literature survey of in vitro and/or in vivo robust experimental data, some common plant-derived alkaloids have dual antiviral and immunomodulatory effects on both RNA and DNA viruses, as well as on inflammatory-related diseases, including cancer and rheumatic disorders [40]. This prompted us to investigate the potential multifocal effects of three structurally representative alkaloids; these are depicted in Figure 1. Their effects were compared to that of the 4-aminoquinoline chloroquine as a reference for antiviral and immunomodulatory effects [41].

Berberine (BER) is an isoquinoline alkaloid derived from various plants, mostly from the genus *Berberis* (Berberidaceae family), with a wide range of biological activities [42,43]. The antiviral potential of BER has been extended to CHIKV, SFV, and SINV [44,45]. Matrine (MAT) is a quinolizidine alkaloid isolated from various plants of the genus *Sophora* L. (Fabaceae family) with biological activities, including anti-inflammatory and antiviral effects, but no specific anti-alphaviral activity has yet been described [46]. Tabersonine (TAB) is an indole alkaloid isolated from the medicinal plant *Catharanthus roseus* (L.) G.Don (Apocynaceae family), which is endowed with potent anti-inflammatory activities [47].

## 2. Results

### 2.1. Cytotoxicity

**Figure 2:**
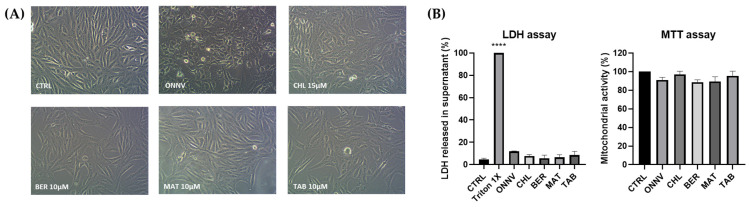
HS633T cell viability: (**A**) microscopy (×200). (**B**) Cells were infected with ONNV or treated with chloroquine (CHL), berberine (BER), matrine (MAT), or tabersonine (TAB) for 24 h. Release of LDH and mitochondrial reductase activity were measured using colorimetric methods. Values are expressed as mean ± SEM of three independent experiments. *p*-value was calculated using the Bonferroni multiple comparison test (****: *p* < 0.0001) when compared with untreated cells (CTRL).

The viability of HS633T cells was assessed under infection by ONNV (MOI 1) or treatment by each of the three studied alkaloids (10 µM) in comparison with CHL (15 µM). The microscopy images (Figure 2A) showed morphological changes in the cells only when they were infected by ONNV. In the LDH and MTT assays, as shown in Figure 2B, no significant LDH release from the supernatants during infection or chemical treatment was detected, and the mitochondrial activity was not significantly affected. Thus, our further experiments were performed using the validated non-toxic concentrations.

### 2.2. Effects of Three Alkaloids on the Viral Replication

**Figure 3:**
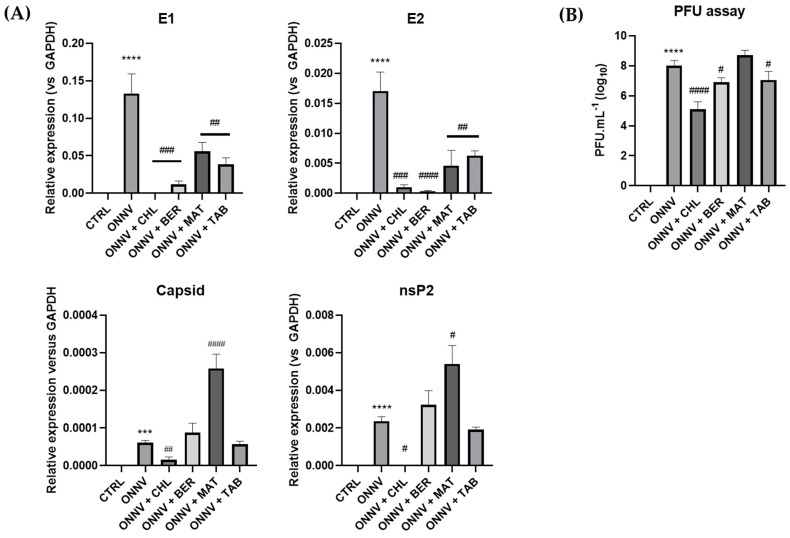
Downregulation of expression of viral genes and reduction in progeny production on HS633T cells. (**A**) HS633T cells were infected with ONNV (MOI 1) and/or co-treated with chloroquine (CHL), berberine (BER), matrine (MAT), or tabersonine (TAB) for 24 h. RNA was collected and viral E1, E2, capsid, and nsP2 gene expression was determined by qRT-PCR analysis. (**B**) Plaque assays were performed on supernatants of the same treated cells. Values are expressed as mean ± SEM of three independent experiments. *p*-value was calculated using the Bonferroni multiple comparison test: ***: *p* < 0.001, ****: *p* < 0.0001 when compared to control cells (CTRL) and #: *p* < 0.05, ##: *p* < 0.01, ###: *p* < 0.001, ####: *p* < 0.0001 when compared to infected cells (ONNV).

We performed qRT-PCR to analyze several RNA coding expressions of the viral proteins during infection alone or together with the different chemical treatments. As shown in Figure 3A, the expressions of all the studied viral RNA were significantly upregulated during ONNV infection (E1: 1.33 × 10^−1^ ± 7.69 × 10^−2^, ****: *p* < 0.0001; E2: 1.70 × 10^−2^ ± 9.83 × 10^−3^, ***: *p* < 0.001; capsid: 6.13 × 10^−5^ ± 3.54 × 10^−5^, ***: *p* < 0.0001; and nsP2: 2.35 × 10^−3^ ± 2.39 × 10^−4^, ****: *p* < 0.0001) when compared to the control cells (CTRL). When compared to the infected untreated cells (ONNV alone), chloroquine (CHL) significantly decreased the expression of all the studied viral genes (E1: 3.76 × 10^−4^ ± 2.17 × 10^−4^, ***: *p* < 0.001; E2: 1.00 × 10^−3^ ± 5.78 × 10^−4^, ***: *p* < 0.001; capsid: 1.60 × 10^−5^ ± 9.23 × 10^−6^, **: *p* < 0.01; and nsP2: 7.57 × 10^−6^ ± 5.62 × 10^−6^, *: *p* < 0.05). Downregulation of the viral E1 and E2 genes occurred with almost all the chemical treatments, but counterintuitively, MAT significantly increased the expression of the capsid and nsP2 genes. The above results were consistent with the significant reduction in the viral progeny production (expressed as plaque-forming units, PFUs) (Figure 3B) observed upon treatment by CHL (8.21 × 10^4^ ± 2.13 × 10^4^ PFU.mL^−1^, ####: *p* < 0.0001); BER ((9.33 × 10^6^ ± 3.53 × 10^6^ PFU.mL^−1^, #: *p* < 0.05); and TAB (1.68 × 10^7^ ± 7.20 × 10^6^ PFU.mL^−1^, #: *p* < 0.05) when compared to the non-treated and infected cells (ONNV).

### 2.3. Effects of Infection and Three Alkaloids on the Antiviral Type-I Interferon Signaling Pathway

The host cell intrinsic antiviral response was first studied from its initiation with the viral receptor MxRa8, the PRR RIG-I, the downstream signals of the IFNAR-dependent pathways (IFN-β), including the IRF3-dependent JAK/STAT pathway, and the resulting production of ISG-15.

**Figure 4:**
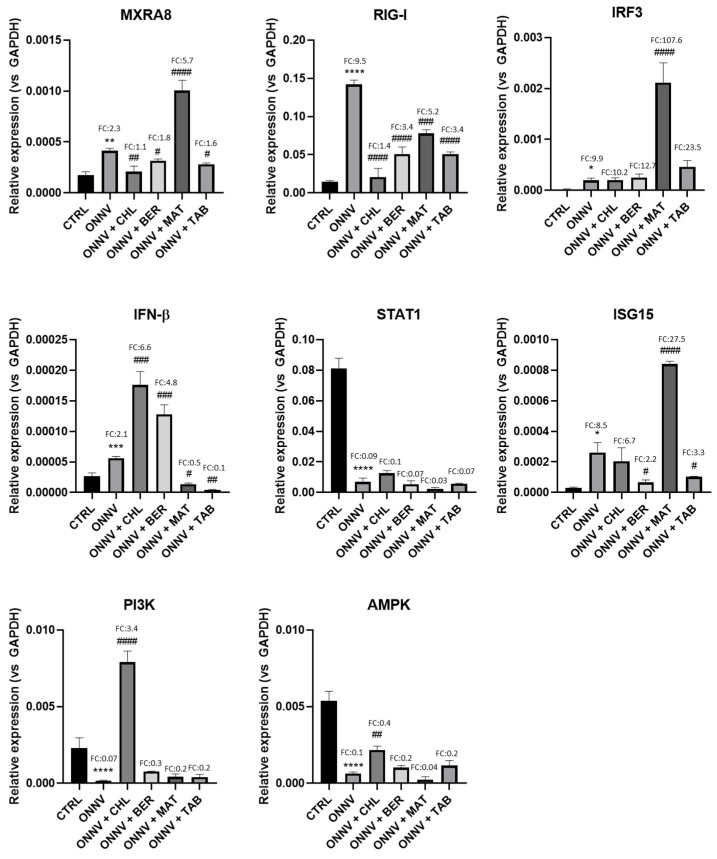
Regulation of the immune antiviral response. HS633T cells were infected with ONNV (MOI 1) and/or co-treated with chloroquine (CHL), berberine (BER), matrine (MAT), or tabersonine (TAB) for 24 h. RNA was collected and the receptors (MxRa8 and RIG-I), interferon regulatory factors (IRF3), signal transducer and activator of transcription 1 (STAT1), antiviral IFN-β and ISG15, and PI3K and AMPK gene expression was determined by qRT-PCR analysis. Values are expressed as mean ± SEM of three independent experiments. *p*-value was calculated using the Bonferroni multiple comparison test: *: *p* < 0.05, **: *p* < 0.01, ***: *p* < 0.001, ****: *p* < 0.0001 when compared to non-infected cells (CTRL) and #: *p* < 0.05, ##: *p* < 0.01, ###: *p* < 0.001, ####: *p* < 0.0001 when compared to infected cells (ONNV).

As shown in Figure 4, the gene expression level of MXRA8 in non-infected cells (CTRL relative to the housekeeping gene GAPDH) was 1.75 × 10^−4^ ± 3.34 × 10^−5^. This expression was significantly upregulated by ONNV infection (4.13 × 10^−4^ ± 2.39 × 10^−5^, **: *p* < 0.01) by a factor (fold change, FC) of 2.3. The RIG-I expression was 1.49 × 10^−2^ ± 1.29 × 10^−3^ in the CTRL and was upregulated (FC: 9.5) in response to ONNV (1.42 × 10^−1^ ± 5.51 × 10^−3^, ****: *p* < 0.0001).

When compared to the infected cells (ONNV), the MXRA8 gene expression was downregulated upon treatment by CHL (2.08^−4^ ± 5.55 × 10^−5^, ##: *p* < 0.01), BER (3.17 × 10^−4^ ± 1.63 × 10^−5^, #: *p* < 0.05), and TAB (2.81 × 10^−4^ ± 1.33 × 10^−5^, #: *p* < 0.05), but it was drastically increased when using MAT (1.01 × 10^−3^ ± 1.00 × 10^−4^, ####: *p* < 0.0001), with an FC that was 3-fold higher when compared to ONNV. The RIG-I gene expression was downregulated by the all the tested compounds (CHL: 2.07 × 10^−2^ ± 1.14 × 10^−2^, ####: *p* < 0.001; BER: 5.09 × 10^−2^ ± 9.29 × 10^−3^, ####: *p* < 0.0001; MAT: 7.81 × 10^−2^ ± 4.88 × 10^−3^, ###: *p* < 0.001; TAB: 5.07 × 10^−2^ ± 2.86 × 10^−3^, ####: *p* < 0.0001).

When compared to the control cells (CTRL, 1.75 × 10^−4^ ± 3.34 × 10^−5^), ONNV increased the gene expression of IRF3 (1.95 × 10^−4^ ± 4.64 × 10^−5^, *: *p* < 0.05; FC: 9.9); IFN-β (CTRL: 2.67 × 10^−5^ ± 5.25 × 10^−6^; ONNV: 5.63 × 10^−5^ ± 2.83 × 10^−6^, ***: *p* < 0.001; FC: 2.1); and ISG-15 (CTRL: 3.06 × 10^−5^ ± 4.75 × 10^−6^; ONNV: 2.61 × 10^−4^ ± 6.47 × 10^−5^, *: *p* < 0.05; FC: 8.5), but it reduced the gene expression of STAT1 (CTRL: 8.11 × 10^−2^ ± 6.70 × 10^−3^; ONNV: 7.00 × 10^−3^ ± 2.39 × 10^−3^, ****: *p* < 0.0001; FC: 0.09).

In comparison with the infected cells (ONNV), we observed a distinct effect of MAT that significantly upregulated the gene expression of IRF3 (2.11 × 10^−3^ ± 3.92 × 10^−4^, ####: *p* < 0.0001) and ISG15 (8.41 × 10^−4^ ± 1.80 × 10^−5^, ####: *p* < 0.0001). The gene expression of IFN-β was upregulated when using CHL (1.76 × 10^−4^ ± 2.17 × 10^−5^, ###: *p* < 0.001) or BER (1.28 × 10^−4^ ± 1.57 × 10^−5^, ###: *p* < 0.001), but it was significantly downregulated by MAT (1.36 × 10^−5^ ± 1.93 × 10^−6^, #: *p* < 0.05) and TAB (4.03 × 10^−6^ ± 1.11 × 10^−6^, ##: *p* < 0.01). CHL and the other alkaloid treatments had no effect on the downregulation of the STAT1 gene expression by ONNV infection.

Autophagy is assumed to be a part of the host cell antiviral defense and is mainly mediated by PI3K/AKT [24]. In our experimental conditions, we observed the downregulation of the gene expression of PI3K (CTRL: 2.29 × 10^−3^ ± 6.84 × 10^−4^; ONNV: 1.63 × 10^−4^ ± 3.90 × 10^−5^, ****: *p* < 0.0001) and AMPK (CTRL: 5.36 × 10^−3^ ± 6.32 × 10^−4^; ONNV: 6.44 × 10^−4^ ± 3.94 × 10^−5^, ****: *p* < 0.0001) by ONNV. From the chemical treatment, only CHL showed a significant upregulation for PI3K and AMPK (7.89 × 10^−3^ ± 7.37 × 10^−4^, ##: *p* < 0.01 and 2.18 × 10^−3^ ± 2.46 × 10^−4^, ##: *p* < 0.01, respectively).

### 2.4. Effects of Infection and Three Alkaloids on Inflammatory Mediators and Signaling Pathways

**Figure 5:**
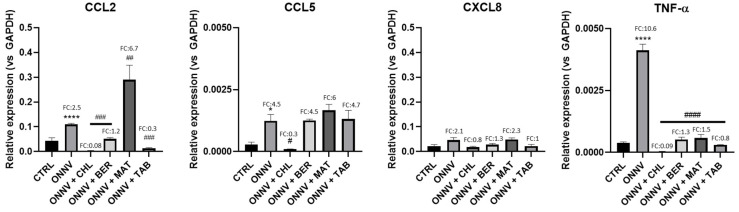
Effect on key inflammatory mediators. HS633T cells were infected with ONNV (MOI 1) and/or co-treated with chloroquine (CHL), berberine (BER), matrine (MAT), or tabersonine (TAB) for 24 h. RNA was collected and CCL2, CCL5, CXCL8, and TNF-α gene expression was determined by qRT-PCR analysis. Values are expressed as mean ± SEM of three independent experiments. *p*-value was calculated using the Bonferroni multiple comparison test: *: *p* < 0.05, ****: *p* < 0.0001 when compared to non-infected cells (CTRL) and #: *p* < 0.05, ##: *p* < 0.01, ###: *p* < 0.001, ####: *p* < 0.0001 when compared to infected cells (ONNV).

The anti-inflammatory potential of the studied alkaloids was first assessed on the key inflammatory mediators. When compared to the CTRL, as shown in Figure 5, the ONNV infection significantly increased the gene expression of CCL2 (CTRL: 4.30 × 10^−2^ ± 1.25 × 10^−2^; ONNV: 1.10 × 10^−1^ ± 3.61 × 10^−3^, ****: *p* < 0.0001; FC: 2.5); CCL5 (CTRL: 2.76 × 10^−4^ ± 1.07 × 10^−4^; ONNV: 1.24 × 10^−3^ ± 2.56 × 10^−4^, *: *p* < 0.05; FC: 4.5); and TNF-α (CTRL: 3.87 × 10^−4^ ± 3.44 × 10^−5^; ONNV: 4.12 × 10^−3^ ± 2.47 × 10^−4^, ****: *p* < 0.0001; FC: 10.6).

When compared to the infected cells, CHL proved to be efficient in decreasing the expression of CCL2 (3.40 × 10^−3^ ± 1.06 × 10^−3^, ###: *p* < 0.001), CCL5 (9.30 × 10^−5^ ± 1.42 × 10^−5^, #: *p* < 0.05), and TNF-α (3.52 × 10^−5^ ± 7.97 × 10^−6^, ####: *p* < 0.0001). The other alkaloids had no significant effect on the regulation of CCL5 expression. Interestingly, BER and TAB exerted substantial downregulation (####: *p* < 0.0001) on both the CCL2 and the TNF-α gene expression. MAT upregulated the gene expression of CCL2 (2.90 × 10^−1^ ± 5.89 × 10^−2^, #: *p* < 0.05), while decreasing the TNF-α level (5.83 × 10^−4^ ± 1.40 × 10^−4^, ####: *p* < 0.0001). None of these treatments significantly affected the gene expression of CXCL8.

### 2.5. Effects of Infection and Three Alkaloids on Inflammatory Signaling Pathways

**Figure 6:**
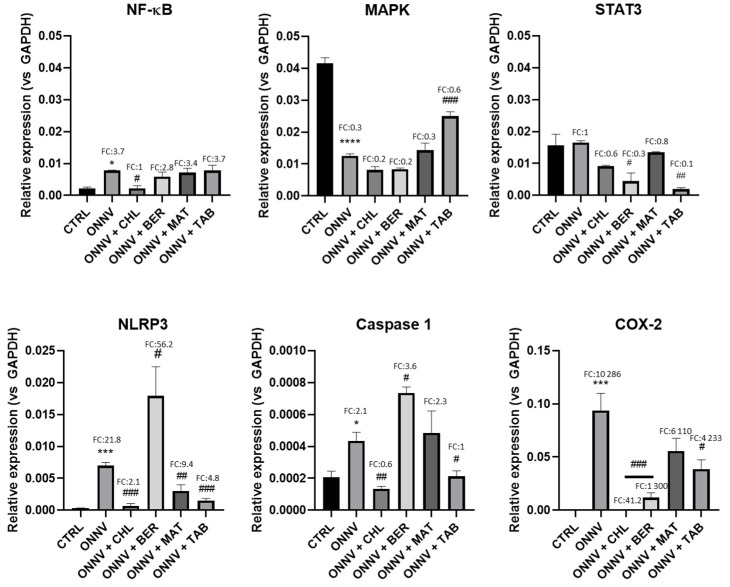
Effect on inflammatory pathways. HS633T cells were infected with ONNV (MOI 1) and/or co-treated with chloroquine (CHL), berberine (BER), matrine (MAT), or tabersonine (TAB) for 24 h. RNA was collected and MAPK, NF-κB, STAT3, NLRP3, caspase-1, and COX-2 gene expression was determined by qRT-PCR analysis. Values are expressed as mean ± SEM of three independent experiments. p-value was calculated using the Bonferroni multiple comparison test: *: *p* < 0.05, ***: *p* < 0.001, ****: *p* < 0.0001 when compared to non-infected cells (CTRL) and #: *p* < 0.05, ##: *p* < 0.01, ###: *p* < 0.001, when compared to infected cells (ONNV).

The above results prompted us to investigate the effects of our alkaloids on the multiple alphaviral-related inflammatory signaling pathways, and the results are presented in Figure 6.

When compared to the CTRL, the ONNV infection increased the gene expression of NF-κB (CTRL: 2.11 × 10^−3^ ± 5.12 × 10^−4^; ONNV: 7.78 × 10^−3^ ± 1.04 × 10^−4^, *: *p* < 0.05; FC: 3.7), which was substantially downregulated by CHL (2.22 × 10^−3^ ± 8.49 × 10^−4^, #: *p* < 0.05). Surprisingly, ONNV downregulated the MAPK gene expression (CTRL: 4.16 × 10^−2^ ± 1.68 × 10^−3^; ONNV: 1.25 × 10^−2^ ± 8.14 × 10^−3^, ****: *p* < 0.0001) when compared to the CTRL, and this effect was significantly attenuated only by TAB (2.50 × 10^−2^ ± 1.37 × 10^−3^, ###: *p* < 0.001). ONNV infection did not affect the gene expression of STAT3 (CTRL: 1.57 × 10^−2^ ± 3.50 × 10^−3^; ONNV: 6.96 × 10^−3^ ± 5.00 × 10^−4^). Interestingly, BER (*p* < 0.05) and TAB (*p* < 0.01) significantly downregulated the STAT3 gene expression when compared to the infected cells (ONNV). ONNV infection upregulated the gene expression of NRLP3 (CTRL: 3.19 × 10^−4^ ± 3.62 × 10^−5^; ONNV: 6.96 × 10^−3^ ± 5.00 × 10^−4^, ***: *p* < 0.001; FC: 21.8) and caspase-1 (CTRL: 2.06 × 10^−4^ ± 3.84 × 10^−5^; ONNV: 4.35 × 10^−4^ ± 5.36 × 10^−5^, *: *p* < 0.05; FC: 2.1), and this upregulation was heightened in the infected cells by BER (NRLP3: 1.79 × 10^−2^ ± 4.58 × 10^−3^, #: *p* < 0.05; caspase-1: 7.35 × 10^−4^ ± 3.67 × 10^−5^, #: *p* < 0.05). MAT only downregulated the NLRP3 gene expression (3.00 × 10^−3^ ± 9.90 × 10^−4^, ##: *p* < 0.01), while TAB downregulated the NLRP3 (1.52 × 10^−3^ ± 3.34 × 10^−4^, ###: *p* < 0.001) and caspase-1 (2.13 × 10^−4^ ± 3.38 × 10^−5^, #: *p* < 0.05) gene expression, as already observed with CHL (NLRP3: 6.70 × 10^−4^ ± 3.87 × 10^−4^, ###: *p* < 0.001; caspase-1: 1.34 × 10^−4^ ± 1.57 × 10^−5^, ##: *p* < 0.01).

The robust upregulation of the COX-2 gene expression (CTRL: 6.28 × 10^−6^ ± 5.66 × 10^−6^; ONNV: 9.37 × 10^−2^ ± 1.63 × 10^−2^, ***: *p* < 0.001; FC > 10 000) by ONNV infection was remarkably suppressed by CHL (3.76 × 10^−4^ ± 1.80 × 10^−4^, ###: *p* < 0.001), BER (1.18 × 10^−2^ ± 4.66 × 10^−3^, ###: *p* < 0.001), and TAB (3.86 × 10^−2^ ± 8.76 × 10^−3^, #: *p* < 0.05).

## 3. Discussion

We surmised the anti-alphaviral potential of the alkaloids by their abilities to control the viral infection and to modulate the complex array of the innate host cell immune response [40]. In this work, we scrutinized and compared the multifocal effects of three pharmacologically relevant plant-derived alkaloids (berberine, matrine, and tabersonine) from three structural classes (isoquinoline, quinolizidine, and indole, respectively) (Figure 1) against the potent reemergent arthritogenic alphavirus ONNV. Our study was performed using HS633T cells to reflect the tropism of arthritogenic alphaviruses for fibroblasts, as reported for CHIKV [14], with non-toxic concentrations of the alkaloid types (10 μM) and the continuously reevaluated antimalarial CHL (15 μM) as potent antivirals against reemerging viruses, at least in vitro (Figure 2).

Our results confirmed the infectivity of HS633T cells by ONNV (MOI 1), as established by the expression of critical viral E1, E2, capsid, and nsP2 genes (Figure 3A). E1 and E2 glycoproteins are essential for virus entry [9,48]. All the non-structural proteins (nsP1-nsP4) are normally required for RNA synthesis [49], but the nsP2 protein is established as a virulence factor and inhibitor of the IFN-induced JAK/STAT pathway [48]. In addition, the essential interactions between the capsid protein and the E1 or E2 protein has also been underlined [50]. We showed that all the studied compounds efficiently downregulated E1 and E2 gene expression during ONNV infection, in spite of a distinct upregulation of capsid and nsP2 gene expression by MAT. Consistently, the viral progeny production was reduced when using BER or TAB (Figure 3B). These results suggest the intracellular effect of the tested compounds, most probably by interference with viral replication.

As revealed by this study, ONNV infection triggers a robust host immune response from HS633T cells. The upregulation of MXRA8 grants effective viral entry [17]. The elevated expression of RIG-I (Figure 4) indicated that the infected cell was able to produce a robust antiviral response [51]. The gene expression of these host cell sensors for viruses was downregulated by BER and TAB, as it was for CHL, but a contrasting upregulation of MXRA8 was observed with MAT and at a level higher than that after ONNV infection. The activation of the type-I IFN antiviral response by HS633T cells was supported by the upregulation of the IFN-β and ISG15 gene expression during ONNV infection, and the studied compounds exerted distinct effects. The counterintuitive effects of MAT were reflected by the upregulation of the IRF3 and ISG15 gene expression, while drastically downregulating the IFN-β gene expression. Using CHL, BER, or TAB, there was no observable effect on the IRF3 gene expression, but there were significant effects on IFN-β and ISG15. BER increased the IFN-β gene expression but decreased the ISG15 gene expression, while TAB suppressed the expression of these two genes. The above observations suggest that the activation of the type-I IFN response occurred independently of IRF3. As also evidenced by our experiments, ONNV may be able to impair the STAT1-related antiviral signaling, as previously described for SINV in vitro [52] or CHIKV [14,53]. The STAT1 silencing during the alphavirus infection resulted from the host cell shutoff caused by the nsP2 protein, which affected the JAK-STAT signaling pathway [54]. None of the tested compounds was able to reinstate the STAT1 gene expression. The PI3K/AKT and AMPK-mediated autophagy has been established as a part of the defensive innate immune response for several Old World alphaviruses, including SFV, RRV, and CHIKV [24], but the pro- or antiviral functions of this intracellular process remain questionable [55,56]. We observed that this process was altered in HS633T cells by ONNV and reinstated only by CHL.

Acute to chronic inflammation is a result of alphavirus infection with mild to severe disorders, as supported for CHIKV [57,58,59,60]. We further observed a robust chemokine/cytokine response during ONNV infection resulting from the upregulation of the critical CCL2 (MCP-1), CCL5 (RANTES), and TNF-α inflammatory mediators (Figure 5). CHL efficiently suppressed the gene expression of CCL2, CCL5, and TNF-α. The common effect of the other compounds was revealed by their remarkable downregulation of TNF-α. Their contrasting effects were observed on CCL2 gene expression, which was efficiently downregulated by BER and TAB but upregulated by MAT.

The analyses of the various inflammatory signaling pathways (Figure 6) revealed the upregulation of NF-κB, NRLP3, caspase-1, and COX-2 gene expression during ONNV infection and a significant mRNA level for MAPK, but our data were inconclusive for STAT3. The involvement of the NF-κB pathway has been demonstrated in mature neurons during SINV infection in vitro [61]. NF-κB is involved in both the type-I IFN response [62] and the inflammatory response [63]. Interestingly, our three alkaloid types had no significant effect on NF-κB gene expression, while CHL induced its downregulation. The MAPK pathway is involved in lifecycle of the cell and the inflammatory responses [64], and it was reported that CHIKV induced MAPK expression in human osteosarcoma (HOS) cells [45]. Its downregulation during ONNV infection was consistent with a previous report for SINV in vitro in human neural progenitor (hNPC) cells [65], and its upregulation occurred only with TAB. The downregulation of STAT3 gene expression was also established in vitro for SINV in hNPC [65], but our data were inconclusive. The NLRP3/caspase-1 pathway favors the strong pro-inflammatory mediator release (IL-18 and IL-1β) and pyroptosis [66] and is related to the severity of the alphavirus infection. The upregulation of NRLP3 and caspase-1 by ONNV was consistent with previous reports on in vivo CHIKV infection [25] and in vitro and in vivo MAYV infection with high levels of IL-1β [26], and we established the remarkable regulatory effect of TAB. Finally, the upregulation of the COX-2 gene expression by ONNV in HS633T cells was consistent with the COX-2-mediated prostaglandin response induced by CHIKV infection in human synovial fibroblasts [22], and was significantly resolved by BER and TAB as well as by CHL.

## 4. Material and Methods

### 4.1. Cell Culture and Virus

The HS633T cells were obtained from the European Collection of Authenticated Cell Cultures (ECACC, Porton, UK, 89050201) and were grown in Minimum Essential Medium Eagle (MEM Eagle, PAN Biotech, Aidenbach, Germany, P0408500), supplemented with 5% decomplemented fetal bovine serum (FBS) (PAN Biotech, Aidenbach, Germany, 3302 P290907), L-glutamine 2 mM (Biochrom AG, Berlin, Germany, K0282), 100 U/mL–0.1 mg/mL penicillin-streptomycin (PAN Biotech, Aidenbach, Germany, P0607100), 1 mM sodium pyruvate (PAN Biotech, Aidenbach, Germany, P0443100), and 0.5 μg/mL fungizone (PAN Biotech, Aidenbach, Germany, P0601001). The cells were maintained in a humid atmosphere at 37 °C with 5% CO_2_ and were allowed to grow until 80–90% confluence. An isolate of O’nyong-nyong virus (ONNV) was obtained from the National Reference Center (CNR arbovirus, Marseille, France) and titrated at 107 PFU.mL^−1^ on Vero cells.

### 4.2. Cell Infection and/or Treatment

The HS633T cells were cultivated in 6- or 96-well plates (100,000 cells/well and 10,000 cells/well, respectively) and maintained at 37 °C in a humid atmosphere with 5% CO_2_. The cells were allowed to grow until 80–90% confluence. The cells were then infected with ONNV to a multiplicity of infection 1 (MOI 1) and/or co-treated with CHL, BBR (Sigma-Aldrich, Darmstadt, Germany, 00900585), MAT (Sigma-Aldrich, Darmstadt, Germany, PHL89730), or TAB (Sigma-Aldrich, Darmstadt, Germany, SMB00452) for 24 h.

### 4.3. Cytotoxicity Assays

Lactate dehydrogenase (LDH) and 3-(4-5-dimethylthiazol-2-yl)-2,5-diphenyltetrazolium bromide (MTT) assays were performed. For the LDH assay, the cells were grown in a 96-well plate (10,000 cells/well); then, they were exposed to CHL (15 µM), BER (10 μM), MAT (10 μM), or TAB (10 μM) for 24 h. The plate was centrifugated (4 mn, 2000 rpm); 50 μL of supernatant was put in a new plate, and 50 μL of reagent from the commercial kit (CytoTox 96^®^Non-Radiaoactive Cytotoxicity Assay, G1782, Promega, Madison, WI, USA, G1782) was added. The reaction was stopped 30 mn later, and the optical density was measured at 490 nm (BIOTEK Cytation 5 imaging reader, Winooski, VT, USA). For the MTT assay, the cells were grown in a 96-well culture plate (10,000 cells/well) and were exposed to CHL (15 μM), BER (10 μM), MAT (10 μM), or TAB (10 μM) for 20 h. Twenty microliters of sterile filtered MTT solution (5 mg/mL) was added to each well. After 4 h, the plate was centrifugated (4 mn, 2000 rpm), the medium was removed, and 200 µL of dimethyl sulfoxide was added to resuspend the formazan crystals. The optical density was measured at 560 nm with a reference at 670 nm (BIOTEK Cytation 5 imaging reader, Winooski, VT, USA).

### 4.4. Quantitative Real-Time RT-PCR (qRT-PCR) Analysis

RNA extractions were realized on HS633T infected and/or treated cells with CHL (15 µM), BER (10 μM), MAT (10 μM), or TAB (10 μM) using the Zymo kit (ZYMO, Ozyme Irvine, CA, USA, R1035). The cells were stabilized with 200 µL of RNA Shield, then lysed with 800 µL of lysis buffer. qRT-PCR analyses were performed using one-step RT-PCR with the SYBR^®^ SENSIFAST Kit (Bioline, London, UK, BIO-76005) and the QuantStudio 5 Real-Time PCR System (Thermo Fisher Scientific, Waltham, MA, USA) in a final reaction volume of 5 μL (1 μL of extracted RNA, 2.7 μL of enzyme mix, and 1.3 μL of primer mix). GAPDH was used as a housekeeping gene. Analyses were performed on the results of three independent experiments. The primers used for qRT-PCR are listed in the following table (Table 1).

### 4.5. Statistical Analysis

The data were expressed as mean ± SEM of the three independent experiments. Statistical analysis was achieved using GraphPad Prism version 6.0 software. The *p*-value was calculated from variance analysis followed by a Bonferroni multiple comparison test.

## 5. Conclusions

**Figure 7:**
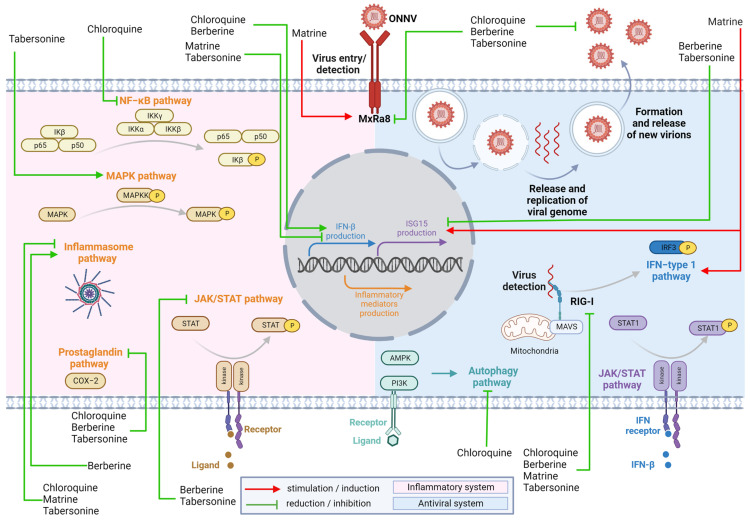
Effect of the studied alkaloids on immune responses (antiviral innate immunity: blue; inflammatory responses: pink). Alphaviruses (e.g., ONNV) use the receptor MXRA8 to bind to and infect fibroblast-like cells of connective tissues. Virus replicates inside the cells and single-stranded viral RNA can be detected by pattern recognition receptors (PRRs) such as RIG-I. Signaling pathways downstream from RIG-I coupled to MAVS activate transcription factors such as IRF3 and contribute to the increased production of type-I IFN (e.g., IFN-β). IFN-β binding to its receptor (IFNAR) activates JAK/STAT pathway and the transcription factors (STAT1/2) to promote the expression of antiviral interferon-stimulated genes (e.g., ISG15 and RNAse L). Viral infection also activates the expression of proinflammatory cytokines (e.g., TNF-α) and chemokines (e.g., CCL2) (notably via NF-κB and MAPK) and prostaglandin expression (via COX-2). The inflammasome contributes to the activation of caspase-1 and increased secretion of the canonical inflammatory cytokine IL1-β. The autophagy response can modulate viral replication and is under the control of two kinases (PI3K and AMPK). The inflammatory response contributes equally, on one hand, to the antiviral response but, on the other hand, if uncontrolled, to chronic injury of the joints. Interestingly, alkaloids (in contrast to the unexpected effect of matrine) control viral infection. Alkaloids can also differentially modulate several steps of proinflammatory responses and open novel therapeutic avenues against alphavirus-induced acute and chronic pathologies. (Figure designed with Biorender).

Our overall results support the paradigm that the ONNV infection of fibroblasts drives a robust antiviral and inflammatory response via the essential type-I IFN pathway and canonical inflammatory signaling pathways (Figure 7). Our findings established the anti-alphaviral potential of the studied alkaloids by the control of viral expansion and the regulation of the host immune response. In-depth biological evaluation of potential antivirals is crucial for their profiling with regard to the immune response, as exemplified by the unexpected behavior of MAT. Finally, these alkaloids may be useful scaffolds for the discovery and development of novel anti-alphaviral drugs.

## Figures and Tables

**Table 1:** List of primers used for qRT-PCR.

Name	Sequence	Supplier
GAPDH	F: CCA TGC GGA AGG TGA AGG TC	Eurogentec
	R: ACA TGT AAA CCA TGT AGT TGA GGT	
E1	F: CAC CGT CCC CGT ACG TAA AA	Eurofins
	R: GGC TCT GTA GGC TGA TGC AA	
E2	F: CCC CTG ACT ACA CGC TGA TG	Eurogentec
	R: CCT TCA TTG GAG CCG TCA CA	
nsP2	F: GCG GAG CAG GTA AAA ACG TG	Eurogentec
	R: TAG AAC ACG CCC GTC GTA TG	
Capsid	F: CGC AGC TTA CGG GTT TCA TA	Genecust
	R: GCA ACG CCT TCA GAA ACG C	
ISG15	F: TTT GCC AGT ACA GGA GCT TGT G	Sigma
	R: GGG TGA TCT GCG CCT TCA	
IFN-β	F: GTC ACT GTG CCT GGA CCA TA	Eurogentec
	R: ACA GCA TCT GCT GGT TGA AGA	
MXRA8	F: TTA CTG TGG CCT GCA CGA AC	Eurogentec
	R: CTC TCG GGG ACG ATG ACA TT	
RIG–I	F: CCA TAT CTC AGC TGG GTG ACA A	Sigma
	R: GCT ATC GGG TCA ACA ACA GCT T	
IRF3	F: CCT CAC GAC CCA CAT AAA ATC	Sigma
	R: GTA GAA GGC TGT CAC CTC GAA	
CCL2	F: CTG CTC ATA GCA GCC ACC TT	Eurogentec
	R: CTT GAA GAT CAC AGC TTC TTT GGG	
IL-1β	F: ACAGATGAAGTGCTCCTTCCA	Eurogentec
	R: GTCGGAGATTCGTAGCTGGAT	
STAT-1	F: TGG TGA AAT TGC AAG AGC TG	Sigma
	R: AGA GGT CGT CTC GAG GTC AA	
MAPK	F: AGCAAGGGAGAGATGGTGTAA	Genecust
	R: CAGTGTCTAAGGGCTGCCAC	
AMPK	F: GGGAAAGTGAAGGTGGGCAA	Genecust
	R: GATGTGAGGGTGCCTGAACA	
NF-κB	CCGGCCCGCCTGAATCATTCTC	Eurogentec
	CAGGTGGCGACCGTGATACCT	
Pi3K	F: ACCATGGAGGAGAACCCTTATG	Genecust
	R: ACGGACAGTGCTCCTCCTTA	
Caspase 1	F: GCTTTCTGCTCTTCCACACC	Genecust
	R: AAATGAAAATCGAACCTTGC	
STAT-3	F: CCGAGCCAATTGTGATGCTT	Genecust
	R: GCATGTTGTACCACAGGATG	
CCL5	F: TCC TCA TTG CTA CTG CCC TC	Eurogentec
	R: TCG GGT GAC AAA GAC GAC TG	
CXCL8	F: CAG AGA CAG CAG AGC ACA CA	Genecust
	R: GGC AAA ACT GCA CCT TCA CA	
TNF-α	F: GCT GCA CTT TGG AGT GAT CG	Sigma
	R: GAG GGG TTT GCT ACA ACA TGG G	

## Data Availability

Data are contained within the article.
